# An Unusual Case of Hepatocellular Carcinoma in a Healthy Adolescent Male

**DOI:** 10.7759/cureus.58357

**Published:** 2024-04-15

**Authors:** Monisha Rita Jayaraman, Lakshmipriya V, Sarah Grace Priyadarshini

**Affiliations:** 1 Department of Pathology, Saveetha Medical College and Hospital, Saveetha Institute of Medical and Technical Sciences, Saveetha University, Chennai, IND

**Keywords:** partial hepatectomy, liver hepatitis hepatocellular carcinoma, cancer in young, liver and gall bladder disease, hepatic tumors

## Abstract

Hepatocellular carcinoma (HCC) is a rare occurrence in adolescents, especially in those without underlying liver diseases. We present the case of a 16-year-old male, having no significant relevant previous medical history, who presented with signs and symptoms of an abdominal mass and hepatic dysfunction. Diagnostic investigations unveiled a startling finding of HCC, challenging the conventional understanding of this malignancy's epidemiology and etiology in young individuals. This example emphasizes the significance of taking HCC into account even in young, healthy individuals who present with unusual symptoms, leading to a comprehensive diagnostic examination and treatment plans customized to meet the specific requirements of patients in their adolescent years. HCC is thought to be more likely to develop in young patients with cirrhosis or fibrosis. The patient in this case study is a young 16-year-old male patient, who was diagnosed with HCC.

## Introduction

Liver cancer is the sixth most commonly diagnosed cancer and the third most common cause of cancer-related deaths worldwide [1]. Generally, infection with the hepatitis B or C viruses is the leading risk factor for hepatocellular carcinoma (HCC) [1]. Hepatitis B is primarily transmitted from mother to child during childbirth in highly endemic regions. Hepatitis B and C viruses can also be transmitted through improper injections and medical procedures as well as less frequently through sexual contact. Aflatoxin consumption, obesity, diabetes, and binge drinking all contribute to HCC. The prognosis for HCC varies depending on factors such as the stage of the cancer, the extent of liver damage, and the effectiveness of treatment. Early detection and treatment can improve outcomes, but HCC is often diagnosed at advanced stages when treatment options may be more limited. Although HCC is thought to account for about 77% of liver cancer cases in the US, there are no reliable estimates on the burden of primary liver cancer by subtype for the entire world [1,2]. Here, we use population-based cancer registry (PBCR) data to offer global, regional, and national estimates of the burden of liver cancer, or HCC. 

## Case presentation

A 16-year-old male came to the outpatient clinic with complaints of abdominal pain and a previously diagnosed history of hepatoblastoma. The patient was admitted to the hospital after which routine blood investigations and image-guided fine-needle aspiration cytology (FNAC) were done. A complete blood count revealed a hemoglobin of 10.4 g/dl, a total red blood cell (RBC) count of 3.40 million/cu.mm, a mean corpuscular volume (MCV) of 89.7 fl, a mean corpuscular hemoglobin (MCH) of 30.6 pg, a mean corpuscular hemoglobin concentration (MCHC) of 34.1 g/dl, a total leucocyte count of 13,260 cells/mm^3^, and a platelet count of 2.21 lakhs/mm^3^. Random blood sugar was normal with a value of 114 mg/dl. Bleeding time and clotting time were measured which were 1 min 43 sec and 3 min 43 sec, respectively. Prothrombin time was 15.6 sec, activated prothrombin time was 35.4 sec, and the International Normalized Ratio (INR) was 1.36. Serum electrolytes were measured which showed serum sodium of 135 mmol/L, serum potassium of 4.4 mmol/L, serum chloride of 105 mmol/L, and serum bicarbonate of 20.1 mmol/L. Renal and liver function tests were performed. Results showed blood urea of 34 mg/dl, serum creatinine of 0.9 mg/dl, serum uric acid of 5.7 mg/dl, total bilirubin of 2.74 mg/dl, direct bilirubin of 0.36 mg/dl, serum glutamic oxaloacetic transaminase (SGOT) of 242 IU/L, serum glutamate pyruvate transaminase (SGPT) of 183 IU/L, alkaline phosphatase of 64 IU/L, total protein of 4.8 g/dl, and albumin of 2.4 g/dl.

**Figure 1:**
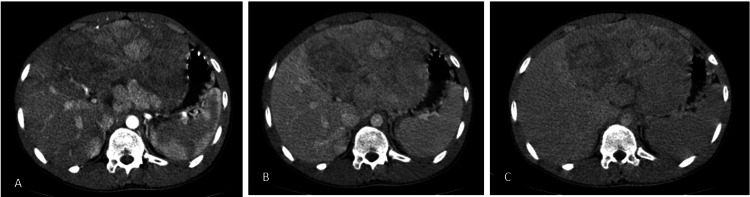
Contrast-enhanced CT abdomen CT: Computed tomography (A) Arterial phase, (B) portovenous phase, and (C) delayed phase images showing heterogeneously enhancing multifocal intraparenchymal and subcapsular lesions involving segments IVA, II, III and IVB of the left lobe of the liver with persistent enhancement and no evidence of washout

Contrast-enhanced computed tomography (CT) of the abdomen was done which showed fairly defined multifocal areas of intraparenchymal and subcapsular nodules of heterogeneous attenuation (predominantly hypodense) lesions involving the left lobe of the liver (Figure 1). No enlarged lymph nodes were noted, and no tumor thrombus identified. An impression of primary malignant neoplasm was given, but the radiological diagnosis pointed more toward hepatoblastoma than HCC.

**Figure 2:**
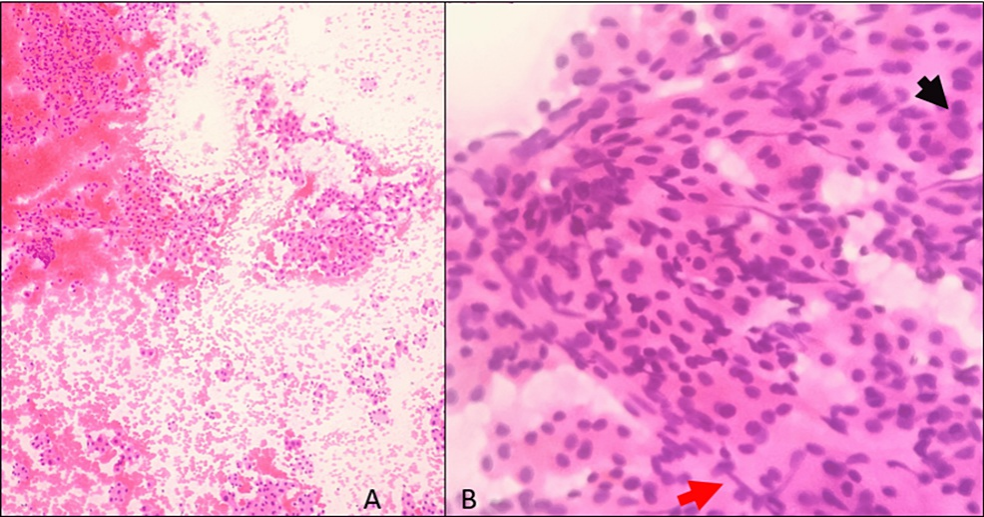
Fine-needle aspiration cytology of the liver lesion (A) Hematoxylin and eosin (H&E) 10X power shows the cellular smear showing sheets, clusters, and singly scattered cells in a background of eosinophilic material and hemorrhage. (B) H&E 40X power shows a higher magnification of the image which shows cells with round to oval nuclei with abundant eosinophilic cytoplasm and mild anisonucleosis (black arrow) with few spindle cells (red arrow)

Considering this, an ultrasonogram-guided FNAC of the let lobe of the liver was done. Cellular smears show sheets, clusters, acinar, papillary pattern, and singly scattered cells having round to oval nuclei with abundant eosinophilic cytoplasm with traversing blood vessels. Few cells show mild anisonucleosis, intracytoplasmic bile, and prominent nucleoli admixed with fibrous strands and spindle cells in a background of eosinophilic material and hemorrhage (Figure 2). Smears were positive for malignancy, and biopsy was suggested for confirmation of diagnosis.

**Figure 3:**
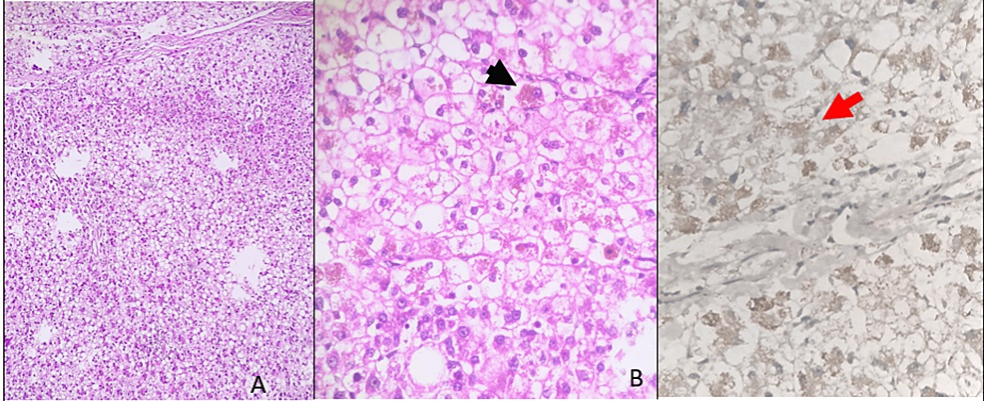
Histopathological examination of the tumor and HepPar1 immunohistochemistry HepPar1: Antihepatocyte-specific antigen (A) Hematoxylin and eosin (H&E) 10X power shows increased trabecular plate thickness, fatty change. (B) H&E 40X power shows a closer look which shows cells that resembled mature hepatocytes with minimal to mild atypia, vesicular nucleus, and prominent nucleolus and few cells showing intracytoplasmic bile (black arrow) and focal areas of clear cell change. (C) Immunohistochemistry 40X power shows HepPar1 granular cytoplasmic positivity (red arrow) of the tumor showing hepatocellular origin

The patient underwent surgery and extended hepatectomy of the left lobe of the liver involving segments II, III, IV-A, and IV-B along with cholecystectomy. We received the formalin-fixed specimens of the same in the histopathology lab. A partial hepatectomy specimen with a solitary tumor of size 14 x 10 x 9 cm was received. Histopathological examination revealed a neoplasm composed of cells arranged in trabecular pattern and sheets. Cells were similar to the appearance of mature hepatocytes with increased trabecular plate thickness, minimal to mild atypia, vesicular nucleus, and a prominent nucleolus. Few cells showed intracytoplasmic bile, and focal areas showed clear cell change (Figure 3). Adjacent liver parenchyma was unremarkable, and an impression of a well-differentiated HCC grade 1 with no regional lymph node metastasis and pathological stage classification of pT3 pN0 was given.

The patient had an uneventful postoperative recovery. Magnetic resonance cholangiopancreatography (MRCP) was done which was normal. The patient was under regular follow-up. Then, the hemogram and liver function tests taken two months after surgery were within normal limits.

## Discussion

Liver cancer develops secondary to a chronic liver disease or environmental factors which can be easily attributed in almost 90% of the cases [1,2]. The various causes of chronic liver disease are viral infections like hepatitis B and hepatitis C and alcohol abuse causing steatohepatitis, and other causes like biliary atresia, progressive familial intrahepatic cholestasis, alpha-1 antitrypsin deficiency, viral hepatitis, HIV infection, liver cirrhosis, metabolic disorders such as tyrosinemia, and glycogen storage diseases are broadly classified under nonalcoholic causes [2-4]. Also included in this category are the environmental factors which comprise a number of exogenous toxins like aflatoxin, a fungal toxin, vinyl chloride, a chemical used in the production of polyvinyl chloride (PVC) plastics, occupational exposure to heavy metals like arsenic and cadmium, organic solvents, N-nitrosamines, insecticide dichloro-diphenyl-trichloroethane (DDT) and to some extent cigarette smoking [1,5]. The remaining 10% comprise sporadic HCC which develops in the absence of any liver disease or infection.

Given the age of the patient, the potential differential diagnoses will be hepatoblastoma, focal nodular hyperplasia, liver abscess, hepatic adenoma, primary hepatic lymphoma, and undifferentiated embryonal sarcoma [2-4]. The two most common histopathological types of HCC in children are classic and fibrolamellar type. Fibrolamellar type is more common in patients who are younger, Caucasian, have no cirrhosis or hepatitis, and alpha-fetoprotein (AFP) within normal limits [2,6]. The clinical profile of our case is somewhat similar, young patient with normal AFP levels and no evidence of cirrhosis or hepatitis, but the histopathological type is classical. AFP has a sensitivity of 41%-65% and specificity of 80%-94% when the cutoff value is 20 ng/ml [7]. So, AFP along with radiological and pathological correlation is more reliable for the diagnosis of HCC. HCC can be unifocal, multifocal, or diffuse on imaging. Smaller lesions are hypoechoic, whereas larger ones are heterogeneous due to fatty change, fibrosis, necrosis, and calcification. Radiologically, multiphasic CT and MRI are considered to be the gold-standard imaging modalities for diagnosing liver cancer [8]. There are case reports of HCC mimicking liver abscess on imaging where patient presented with fever and leukocytosis [3]. But in this case, the patient had elevated AFP levels and no hepatitis or cirrhotic background with microscopy showing evidence of classical HCC. Viral markers were done in view of elevated liver enzymes, which turned out to be negative. Histopathologically, the adjacent liver parenchyma did not show any evidence of hepatitis. The patient did not give any significant drug history, and no further workup for autoimmune causes were done. Finally, the age of the patient and the nonspecific symptoms during presentation makes the case an interesting one.

## Conclusions

Juvenile HCC is typically more advanced and aggressive than adult HCC; thus, as soon as it is diagnosed, quick risk stratification and the start of appropriate therapy are advised. HCC in young individuals without obvious risk factors is an exceptionally rare occurrence, despite the fact that HCC is highly prevalent throughout the world. It is essential to consider HCC while diagnosing space-occupying lesions in the liver of young patients, particularly in regions with high viral hepatitis B and C prevalence.
